# American Academy of Dental Science

**Published:** 1892-07

**Authors:** 

**Affiliations:** American Academy of Dental Science


### AMERICAN ACADEMY OF DENTAL SCIENCE.

The regular monthly meeting of the American Academy of Dental Science was held in the Boston Medical Library Association rooms on February 3, 1892, President Brackett in the chair.

The paper of the evening was read by Thomas Stellwagen, M.D., D.D.S., of Philadelphia; subject, "The Teeth and the Feet."

(For Dr. Stellwagen's paper, see page 502.)

### DISCUSSION.

*Dr. Stellwagen.*—In examination of patients at Orthopædic Hospitals, in nearly every case where the dental irregularity was slight or entirely absent the children had, to a great extent, supplemented by their hands the use of their feet, and thus their mouths were kept in a tolerably healthful condition.

A study of a series of heads showing the development of the teeth demonstrates that at the age of the eruption of the permanent teeth the jaws increase very materially. Hence at this period it is of the utmost importance that the maxillæ should be exercised.

In the list of cases examined there were twenty-four boys and twenty-two girls. Among the boys there were fifteen irregular cases requiring operation for regulating the teeth, some of these also requiring an expansion of the jaw. Of the very irregular cases, there were seven requiring operations both to straighten the teeth and to expand the arch. In twenty-two cases of girls, eight normal cases were found. Six were slightly irregular, eight decidedly irregular.

The cases examined were nearly all in hospitals, and the average age eleven or twelve years.

*Dr. Williams.*—A long time ago the remark was made in one of our best books, "Let not the hand say unto the foot, I have no need of thee," and although that is not the title of Professor Stellwagen's paper, his application of that idea is founded on solid principles.

Another point, too, that struck me in his address was the fact that a corn on the toes or a pinching boot will take away one's appetite, and I can see how, if the irritation exists sufficiently long, a discomfort of this kind would create malnutrition, and its consequent effects would be apt to show themselves quite as much in the teeth as in any other part of the body.

*Dr. Eames.*—This paper is a strong argument that the dentist be medically educated, in order to better understand the significance of the relations existing between different parts of the body.

From the better medical education of the dentist may come important light upon the subjects of erosion, phagedenic pericementitis, and stomatitis.

*Dr. Barker.*—Professor Stellwagen's paper is a new statement of the old fact that function determines form. The moral contained in the paper might well be applied to many of our school-children, whose time is so largely occupied in the translation of Latin and the study of Greek roots that they neglect physical development. Parents who have young children should turn them out to pasture, treat them like colts.

*Dr. Stellwagen.*—My opinion is that the foot should be covered with something more like a moccasin than a shoe, and that children should be allowed to run barefooted as much as possible.

The benefits derived from this practice were demonstrated last summer during a six weeks' sojourn at the sea-shore. Two of our youngest children were allowed to walk and run in the sand without shoes or stockings. The result was that the boy, who is now thirteen, increased in chest girth from sixty to seventy centimetres, with the power of expanding the chest to eighty; so that he really increased his measurement from one-sixth to one-third.

His limbs also were materially changed, the muscles were firmer and more solid, and his foot increased about a size and a half. Would it not be better for children if barefooted exercise were substituted for the use of donkeys and many other means of amusement? Would we not have a better development and a firmer condition of the teeth and jaws as well as of all other parts of the body.

WILLIAM H. POTTER, D.M.D.,
*Editor American Academy of Dental Science.*

